# Harlequin ichthyosis: A case report of severe presentation in Eritrea

**DOI:** 10.1002/ccr3.3076

**Published:** 2020-06-28

**Authors:** Zemichael Ogbe W, Tarig Gasim Mohamed Alarabi

**Affiliations:** ^1^ Department of Paediatrics and Child Health OCMHS Asmara Eritrea; ^2^ Unit of Anatomy and Embryology OCMHS Asmara Eritrea

**Keywords:** *ABCA12* gene mutation, autosomal recessive, congenital skin abnormalities, harlequin ichthyosis

## Abstract

The severe form of harlequin ichthyosis is often lethal in the perinatal period, and it is commonly a product of consanguineous parents. Therefore, in vitro fertilization and pregenetic diagnosis are recommended to avoid the recurrence of the error.

## Graphical abstract

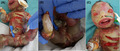

## 1 INTRODUCTION

Harlequin ichthyosis is the most severe form of autosomal recessive congenital ichthyosiform dermatoses, and it primarily affects the skin.^1^ Other systems may be significantly compromised by the hyperkeratosis and concomitant deformities. HI is characterized by a profound thickening of the keratin layer in fetal skin. The affected neonate is born with a massive, horny shell of dense, plate‐like scale and contraction abnormalities of eyes, ears, mouth, and appendages.

The affected newborns commonly die due to acquiring infection secondary to the deep skin fissures and respiratory failure due to restricted respiration by the skin, which impedes the chest wall from expanding and drawing in enough air. Histologically, it is characterized by the existence of extracellular lipid material in the stratum corneal layer of the epidermis.^2^

Harlequin ichthyosis is an inherited autosomal recessive disorder, it affects 1 in every 300.000 neonates, it is triggered by mutations in the *ABCA12* gene (adenosine triphosphate‐binding cassette *A12*), the controlling gene of the developing skin, and in such cases, the (relative marriages) consanguinity is considered as a generating factor for this mutation.^3^

## 2 CASE REPORT

**Figure 1:**
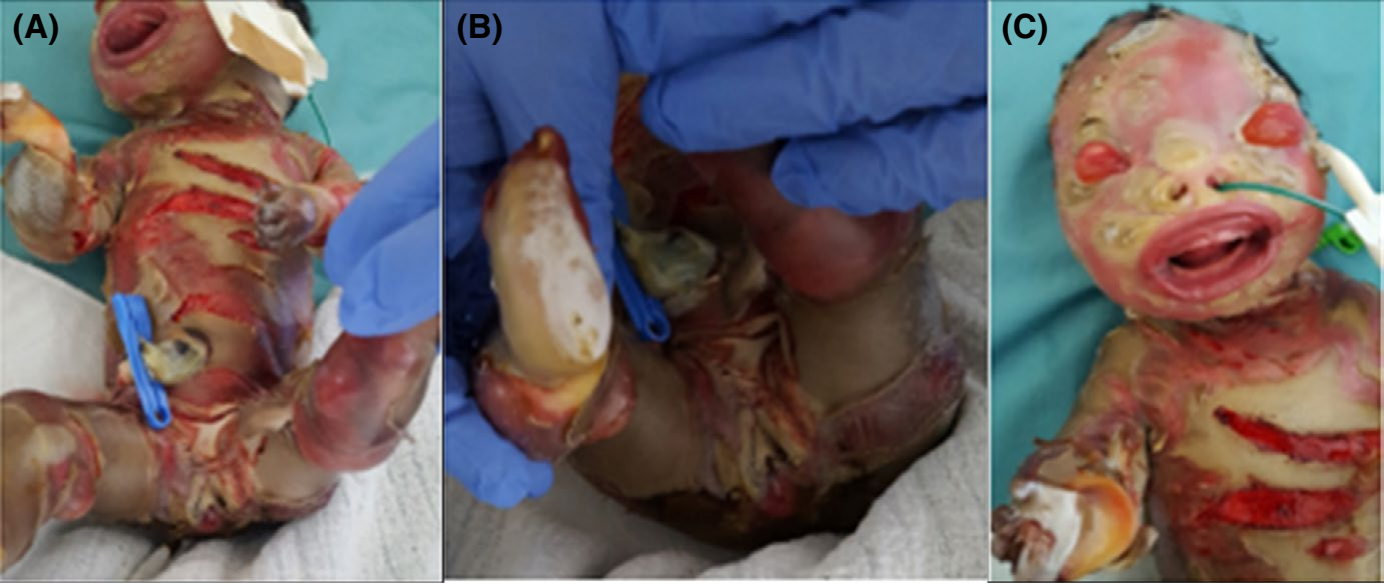
Physical presentation of female newborn diagnosed with severe form of harlequin ichthyosis, A, shows deep skin fissures, B, scaled external genitalia, buttocks and dry, thick sole of foot, and C, shows face with ectropion, eclabium, and right hand with contracture

A 26 years old pregnant woman, blood group B negative, admitted to the Orotta maternity hospital, full term. The pregnant was gravida 4, para 4, A/B 0 with 1 still birth (not similar to current condition). The pregnant was not on regular antenatal follow‐up, and she does not remember her last menstrual period (unknown LMP). She delivered via normal vaginal delivery—the second stage on 07/03/2019, and the amnio‐chorionic membrane was ruptured 45 minutes before the delivery. The neonate (Figure 1) was female, and she was diagnosed with harlequin ichthyosis. Her birthweight was 2.9 kg, temperature: 35.5 C with an Apgar score of 6 at 1st minute and 5th minute, respectively. Parents were in close consanguinity (cousins), and they have no similar condition of harlequin ichthyosis in the previous pregnancy nor family history. They have three healthy children.

The neonate had perinatal asphyxia soon after birth and presented with severe scaled skin interrupted by deep fissures with dry and thick edges affecting her palms and soles by contractures. Hyper keratinization and erythroderma were detected over the entire skin. The head was characterized by a scalp with partial hair loss (Alopecia). Ectropion and eclabium were detected in the face. The limbs were characterized by underdevelopment of fingers and toes (Figure 1). Restricted chest movement was noticed. She was immediately referred to nursery unit (Specialized Neonatal Care Unit—SNCU of Orotta Hospital in Asmara, Eritrea), where she was placed in a humidified incubator to obtain the optimal body temperature and orogastric tube (OGT) was inserted for feeding expressed breast milk.

In view of the imminent infection, a combination of oral antibiotics (amoxicillin and cefuroxime syrups) was given, as IV line was not possible because of severe skin damage. For skin treatment, vitamin A via OGT and Salbe cream (a mix of zinc oxide and lebetran) was administered. She died on 13.03.2019 (5 days after birth).

## 3 DISCUSSION

The first case report of a similar disorder was presented by Hart in South Carolina, United States of America, at 1750.^4^ The current case is considered as the third condition that registered in Eritrea since the first one in 2012. The young mother age is not absolutely a preventing factor from harlequin ichthyosis especially in the presence of close relative marriages (consanguinity),^3^ and a similar condition of female neonate was born to a 22‐year‐old (young mother) primigravida at 33 weeks of gestation were reported.^5^ Meanwhile, the consanguinity it can express the mutation at any age and that agreed with that study which was reported,^1^ a 31‐year‐old pregnant woman from Iran in her third pregnancy at 30 weeks and 1 of gestational age. Also, a case was reported^6^ for a 37‐year‐old (old mother) para 2 + 0, delivered a female infant at 35 weeks + 6 days, with Apgar score of 9, whereas her weight was 3.14 kg. Another case was reported,^7^ it was a male neonate from infertile couples (8 years of infertility), relatives (first degree) born by assisted reproductive technologies (ART) at the 35th week via cesarean section, and his weight was 2500 kg.

## 4 CONCLUSION

The HI neonates could be delivered via any way, from any populations, fertile or infertile couples, different neonate sex, varied weight from normal to underweight, preterm to full term, young or adult to old pregnant, and primigravida to the tertiary one. But most cases were merged to meet on the same causative factor, the consanguinity. In the case of consanguinity marriage, the genetic analysis is recommended to avoid recurrence of the condition especially in the presence of a family history of similar conditions. Also, assisted reproductive technologies (ART) that followed by pregenetic diagnosis (PGD) are recommended for any pregnancy of relatives especially for first degree couples.

## CONFLICT OF INTEREST

None declared.

## AUTHOR CONTRIBUTIONS

ZOW: obtained the patient data used in the review. TGMA: wrote the case report.
